# RNA-Sequencing of *Drosophila melanogaster* Head Tissue on High-Sugar and High-Fat Diets

**DOI:** 10.1534/g3.117.300397

**Published:** 2017-11-15

**Authors:** Wayne Hemphill, Osvaldo Rivera, Matthew Talbert

**Affiliations:** Department of Biology, University of Louisiana at Monroe, Louisiana 71209

**Keywords:** obesity, fly, transcriptome, obesogenic

## Abstract

Obesity has been shown to increase risk for cardiovascular disease and type-2 diabetes. In addition, it has been implicated in aggravation of neurological conditions such as Alzheimer’s. In the model organism *Drosophila melanogaster*, a physiological state mimicking diet-induced obesity can be induced by subjecting fruit flies to a solid medium disproportionately higher in sugar than protein, or that has been supplemented with a rich source of saturated fat. These flies can exhibit increased circulating glucose levels, increased triglyceride content, insulin-like peptide resistance, and behavior indicative of neurological decline. We subjected flies to variants of the high-sugar diet, high-fat diet, or normal (control) diet, followed by a total RNA extraction from fly heads of each diet group for the purpose of Poly-A selected RNA-Sequencing. Our objective was to identify the effects of obesogenic diets on transcriptome patterns, how they differed between obesogenic diets, and identify genes that may relate to pathogenesis accompanying an obesity-like state. Gene ontology analysis indicated an overrepresentation of affected genes associated with immunity, metabolism, and hemocyanin in the high-fat diet group, and CHK, cell cycle activity, and DNA binding and transcription in the high-sugar diet group. Our results also indicate differences in the effects of the high-fat diet and high-sugar diet on expression profiles in head tissue of flies, despite the reportedly similar phenotypic impacts of the diets. The impacted genes, and how they may relate to pathogenesis in the *Drosophila* obesity-like state, warrant further experimental investigation.

Obesity is a low-grade inflammatory condition characterized by a positive energy imbalance, resulting from overabundant energy intake, and/or inadequate energy expenditure. The most commonly used assessment of obesity is the Body Mass Index (BMI) score, which is calculated as weight in kilograms divided by the height in meters squared. Obesity is characterized by the World Health Organization as a BMI > 30, whereas a BMI of 25–30 characterizes being overweight (Lehnert *et al.* 2013). By this metric, 60% of adults in the US population are estimated to be overweight, 30% of which are estimated to be obese (“Adult Obesity Facts|Overweight and Obesity|CDC” n.d.). Obesity’s detriment to health is largely attributed to its facilitation of comorbidities such as cardiovascular disease, type-2 diabetes, and neurological decline. Conditions such as these are not only increased in prevalence, but also exacerbated, in obese individuals (Must *et al.* 1999; Langdon *et al.* 2011). Obesity fosters a molecular environment conducive to comorbidities, and so that environment, and the mechanisms by which it does induce disease, are of great biomedical interest.

While some mechanisms of pathogenesis leading to comorbidity in an obesity or obesity-like state in mammals remain unknown, studies with mice and humans have revealed increased inflammation, aberrant cell signaling, decreased insulin sensitivity, and increased oxidative stress to be involved (Xu *et al.* 2003; Furukawa *et al.* 2004; Montague *et al.* 1997). The elevated release of pro-inflammatory cytokines is characteristic of the inflammatory state in obesity, which can be initiated by accumulation of lipids in adipocytes, retardation of autophagy, and the unfolded protein response, and is characterized by the infiltration of visceral adipose tissue by macrophages (Gregor and Hotamisligil 2011). Pro-inflammatory cytokines such as tumor necrosis factor α (TNFα), interleukin-6 (IL-6), and resistin can promote insulin resistance that can, in turn, be ameliorated by a decrease in visceral adipose tissue (Piya *et al.* 2013). Leptin resistance has been observed in mice following prolonged exposure to a high-fat diet, and this has been hypothesized to further exacerbate positive energy imbalance (Lin *et al.* 2000). Mice subjected to high-fat diets have been shown to have an increase in reactive oxygen species (ROS), indicative of oxidative stress (Furukawa *et al.* 2004).

The effects of an excess energy imbalance on overall longevity are conserved in a range of model organisms (Lee *et al.* 2017). Furthermore, the adipocyte, which is represented in the majority of mammals, as well as flies, is now recognized for its wide range of endocrine effects on organismal homeostasis, in addition to its nutrient storage function (Rodríguez *et al.* 2015). Implicated in this endocrine functionality in the model organism *Drosophila melanogaster* are several conserved hormone functions, including the fat-body-secreted Upd2 (Unpaired 2), which is a leptin ortholog, adipokinetic hormone (Akh), and DILP’s (*Drosophila* insulin-like peptides). Akh is a glucagon ortholog released from the corpora cardiaca (present just posterior the fly head, and often collected during head removal), while DILP2 is an insulin ortholog released from insulin-producing cells (IPCs) in the fly brain (Géminard *et al.* 2009). DILP2 and Akh act in regulating nutrient storage and release in the fly, much like the regulation of fed and fasting states in mammals by insulin and glucagon (Géminard *et al.* 2009).

In *D. melanogaster*, a physiological state mimicking diet-induced obesity can be achieved by subjecting flies to diets high in sugar (HSD), or diets high in fat (HFD). Coconut oil in the HFD, and disproportionately high amounts of sucrose in the HSD, have been shown to be effective in promoting this state (Rovenko *et al.* 2015; Heinrichsen *et al.* 2013). Flies reared on such diets present with increased circulating glucose levels, increased triglyceride content, decreased DILP2 (insulin ortholog) sensitivity, decreased stress tolerance and lifespan (Heinrichsen and Haddad 2012; Heinrichsen *et al.* 2013), and behavior indicative of neurological decline, such as impaired climbing ability (McNay *et al.* 2010; Stranahan and Mattson 2011; Valladolid-Acebes *et al.* 2011; Feany and Bender 2000; Bouleau and Tricoire 2015). Oxidative stress is also a noted consequence of a high-fat and high-sugar diet in the fly (Musselman *et al.* 2011; Heinrichsen *et al.* 2013).

It is also feasible for conserved mechanisms of obesity pathogenesis to exist between flies and mammals given the physiological parallels, that over 75% of human disease-associated genes have orthologs in the *Drosophila* model (Piya *et al.* 2013; Reiter *et al.* 2001), and that almost half of all *Drosophila* genes have functional orthologs in humans (Shih *et al.* 2014). The fly has susceptibility to obesity-related cardiac abnormalities reminiscent of the effect observed in mammals, and an inflammatory response characterized primarily by the production of antimicrobial peptides (AMPs) and recruitment of hemocytes (the fly’s immune cells) (Shaukat *et al.* 2015). The AMPs are a natural part of fly’s humoral immune response to infection, but there is also of evidence of heightened levels of these AMPs having adverse effects on neural tissue, as well as promoting autoimmunity in the fly (Shaukat *et al.* 2015).

Sparing experimental evidence suggests subtle differences accompany the otherwise similar effects of diets high in sugar *vs.* fat. In flies, HFDs have illustrated the ability to cause cardiac abnormalities in the form of increased cardiac triglycerides, decreased and/or absent contractile ability in portions of the heart, and dysfunction of flow valves, among other secondary symptoms (Birse *et al.* 2010). Similarly, HSDs have been shown to cause heart deterioration and arrhythmia in the fly model, in addition to the shortened lifespan and DILP insensitivity caused by both diet types (Na *et al.* 2013). In humans, study of the persisting metabolite and hormone levels following consumption of isocaloric diets higher in fat or sugar has illustrated such differences (Lewis *et al.* 1977).

The Database for Annotation,Visualization, and Integrated Discovery (DAVID) Bioinformatics Resources software allows one to analyze the prevalence of certain functional classes of genes within a provided gene list (Dennis *et al.* 2003). It utilizes information from multiple other databases, like FlyBase (Gramates *et al.* 2017), to provide the functional keyword(s) (called annotations) associated with the genes of a given species. In addition to providing individual annotations of genes, the software can group annotations that are related, or of a similar nature, producing annotation clusters. This relation is gauged based on the number of shared genes between a group of annotations for a specific gene list. This tool can be used on the results of many assays, including RNA-Sequencing.

Our objective was to identify gene expression patterns of interest in the head tissues of fruit flies exposed to a HSD, HFD, or a normal diet (ND) via poly-A selected RNA-Sequencing, and to establish differences between the physiological effects of two conventional obesogenic diet types (HSD and HFD) through expression profiling and gene ontology (DAVID). Furthermore, we hoped to determine the functional nature of genes affected by these diets, with the goal of ascertaining pathogenic mechanisms accompanying obesity in flies, and, by extension, possibly in mammals. Heads were used since they contain the brain, glia, neurosecretory cells (including insulin-like peptide-producing cells, and potentially the corpora cardiaca, which can accompany removal of the head), a pericerebral fat body, and, therefore, many of the components of central energy homeostasis. This approach aimed to reduce competing local transcription patterns produced by the rest of the fly tissues, while still accounting for many of the major tissues involved with metabolism and obesity.

## Materials and Methods

### Flies/rearing

All flies used in the experiment were wild-type, Oregon R-C (Bloomington stock 5) genotype. Upon eclosing, female virgins were collected and housed on low calorie media at approximately equal densities of 30 flies/vial until in excess of 450 flies were obtained. Females were then synchronously mated with males of the same genotype in a 2:1 ratio for 2 d. After mating, males were removed, and the females were divided equally into three groups, and placed on one of the three corresponding media types (ND, HSD, or HFD). The fly groups were kept on their respective medium for 7 d, being transferred to fresh medium every 3–4 d. Flies were kept in an incubator at 25° on a 12-hr light/dark cycle with consistent humidity, until decapitation, excluding any handling without anesthesia for media transfers.

### Media

The ND acted as our control diet, and consisted of 2.6% w/v cornmeal, 4% w/v dry inactivated yeast, 0.8% w/v agar, 3% w/v sucrose, 1.5% v/v Tegosept (20% w/v in 70% ethanol), and 0.3% v/v propionic acid. The HFD and HSD were identical to the ND, except for an addition of 20% w/v coconut oil in the HFD, and the use of 20% w/v sucrose instead of 3% in the HSD. The low calorie (LC) diet briefly used for collections and mating contains 5.2% w/v cornmeal, 5% w/v dry inactivated yeast, 1% w/v agar, 3% w/v sugar, 1.5% v/v Tegosept (20% w/v in 70% ethanol), 0.3% v/v tetracycline (10 mg/ml), and 0.3% v/v propionic acid.

### RNA extraction

Flies were transferred from their media briefly into empty vials and anesthetized using FlyNap(Carolina). The flies were then submerged in a Petri dish with 1 ml TRIzol reagent, and their heads were pulled off with forceps in rapid succession. The heads (*N* = 20) were then immediately placed in 200 µl of TRI Reagentin a 1.5-ml microcentrifuge tube. There were six biological replicates (tubes) for each media group, with each replicate/tube containing 20 heads. The head tissue in each tube was then homogenized using an electric pestle. RNA extraction and purification was performed using a Direct-zol RNA MicroPrep kit (Zymo Research). The RNA was eluted with sterile water, after which purity and concentration were confirmed via 260/280 and 260/230 ratios measured using a NanoDrop 2000c (Thermo Scientific). *N* = 6 per experimental condition.

### Sequencing

Twelve RNA samples (four replicates from each of three dietary treatment groups) of the highest yield (>100 ng/µl) and sufficient purity (260/280 and 260/230 ratios of 1.8–2.1 and >1.5, respectively), ascertained via NanoDrop2000c (Thermo Scientific) readings, were sent to Louisiana State University Health Science Center in Shreveport for initial Poly-A selected RNA-sequencing in the Genomics Core Facility. mRNA was isolated from the RNA via poly-adenylated RNA selection using oligomer beads, and sequenced using a NextSequation 500 (Illumina), targeting at least 50 million stranded, paired-end reads of 75 bp in size. Raw sequencing data were passed through a software filter (RTA v2.4.6.0), and sent to the laboratory of U. Cvek at Louisiana State University at Shreveport for further analysis to ascertain reads per million, perform differential expression analysis between the three conditions, generate the preliminary transcript statistics (discussed later), and identify the sequenced transcripts via mapping against the *Drosophila* mRNA records using Cufflinks (Trapnell *et al.* 2010).

### Statistical analysis

With RNA-Sequencing, the degree of gene expression is gauged (in one case) by a signal value in Reads Per Kilobase per Million reads (RPKM). This is calculated as the number of reads (usually of fragments of comparable size) identifying the transcript, divided by the length of the known whole transcript in kilobases, and divided again by the total number of reads (in millions) for all sequenced transcripts in the sample. Another metric used to illustrate the relative change in RPKM of a transcript between two groups is fold change (FC), which is calculated as the negative log base two of the experimental to control RPKM ratio.

*P*- and *q*-values were also generated, and they were applied to each transcript, in each obesogenic diet group. After obtaining a transcript’s RPKM values for all replicate samples in each diet group, the SD of each of these sample data sets was calculated, and that value applied to a normal distribution of that transcript’s RPKM values for each diet group. These distributions were then compared between the ND and HSD groups, as well as between the ND and HFD groups, and a *p*-value calculated for each obesogenic diet group, in all cases via Cuffdiff (Trapnell *et al.* 2010). *Q*-values were calculated using the *p*-values of each transcript, in each obesogenic diet group (relative to the ND group), via the Benjamini-Hochberg False Discovery Rate (B-H FDR) adjustment method (Benjamini and Hochberg 1995). Prism includes an iteration of this *q* value when performing a B-H FDR adjustment on a set of *p*-values; it indicates the fraction of Type I errors present among all *p*-values less than or equal to its associated *p*-value. Prism 7.0c (GraphPad) was used for all necessary calculations involved with the B-H FDR adjustment.

### Heat maps

Heat maps were created using Heatmap Generator software (Khomtchouk *et al.* 2014). Transcript data were arranged in Microsoft Excel, and the data were then subdivided into six groups, corresponding to the six possible comparative iterations of the RPKM values for each media type (*i.e.*, RPKM values of ND>HSD>HFD, or any other arrangement, for each implicated transcript). A standard normal distribution is generated for each transcript using the RPKM values of that transcript from each diet group, and then a z-score for each diet groups’ RPKM value on that distribution for the transcript is calculated. This z-score is then used to assign a color to that transcript for each diet group, with the most positive z-scores (those with RPKM values above the transcript’s average RPKM value across all diet groups) being bright red, the most negative (those with RPKM values below the transcript’s average RPKM value across all diet groups) being bright green, and anything between being a varied-ratio mix of the two colors.

### Functional annotation

Functional annotation was performed using DAVID Bioinformatics Resources Version 6.8 (Dennis *et al.* 2003). Six gene lists were constructed to be provided, consisting of transcripts with *q* < 0.05 and FC > 0 HFD group values, *q* < 0.05 and FC < 0 HFD group values, *q* < 0.05 and FC > 0 HSD group values, *q* < 0.05 and FC < 0 HSD group values, *q* < 0.05 and FC > 0 HFD and HSD group values, and *q* < 0.05 and FC < 0 HFD and HSD group values, respectively. Each gene list was then separately provided to the software and annotated.

For functional annotation of this data, the “Official Gene Symbol” identification was used, and the species background used was “*D. melanogaster*.” The “Functional Annotation Clusters” feature was used to generate groupings/clusters of related annotations, and their total and individual enrichment values within each gene list. The “Functional Annotation Chart” feature was also used to generate lists of the top individual annotations for each gene list, and their *p*-values. For both features, DAVID Bioinformatics Resources suggested defaults were used for all options, including the database sources, background, and the nature of annotations used, as well as all statistical parameters for determining annotation clusters.

### Functional elaboration of select genes

Genes with *q* < 0.05 in at least one obesogenic diet group were first identified. Those genes were then divided into three lists: those with *q* < 0.05 in both obesogenic diet groups, those with *q* < 0.05 in the HSD group only, and those with *q* < 0.05 in the HFD group only. Each of those gene lists was then separately provided to the DAVID bioinformatics software (Dennis *et al.* 2003), and the genes contained within the most enriched, relevant (potential implications in obesity-related pathogenesis; relating to immunity, metabolism, neural function, *etc*.) annotations were investigated in FlyBase (Gramates *et al.* 2017) for literature supporting functions with experimental evidence. All of these genes were initially considered for elaboration, but those that did not have relevant functions (*i.e.*, functions potentially related to such pathogenesis) were removed from the final list of genes to be discussed; 10 genes were presented with *q* < 0.05 in both obesogenic diet groups, and six genes presented from each of the other two lists.

### Data availability

All data that was generated or analyzed during this research is present in this publication, or its corresponding supplemental files. Raw sequencing data can be accessed on the National Center for Biotechnology Information (NCBI) Gene Expression Omnibus via accession number GSE104336. Supplemental files include comparisons of the expression levels for each gene between the ND and HFDs (Supplemental Material, File S1) and between the ND and HSDs (File S2). RNA-sequencing quality control information is available in File S3.

## Results

### Alterations in gene expression

**Figure 1:**
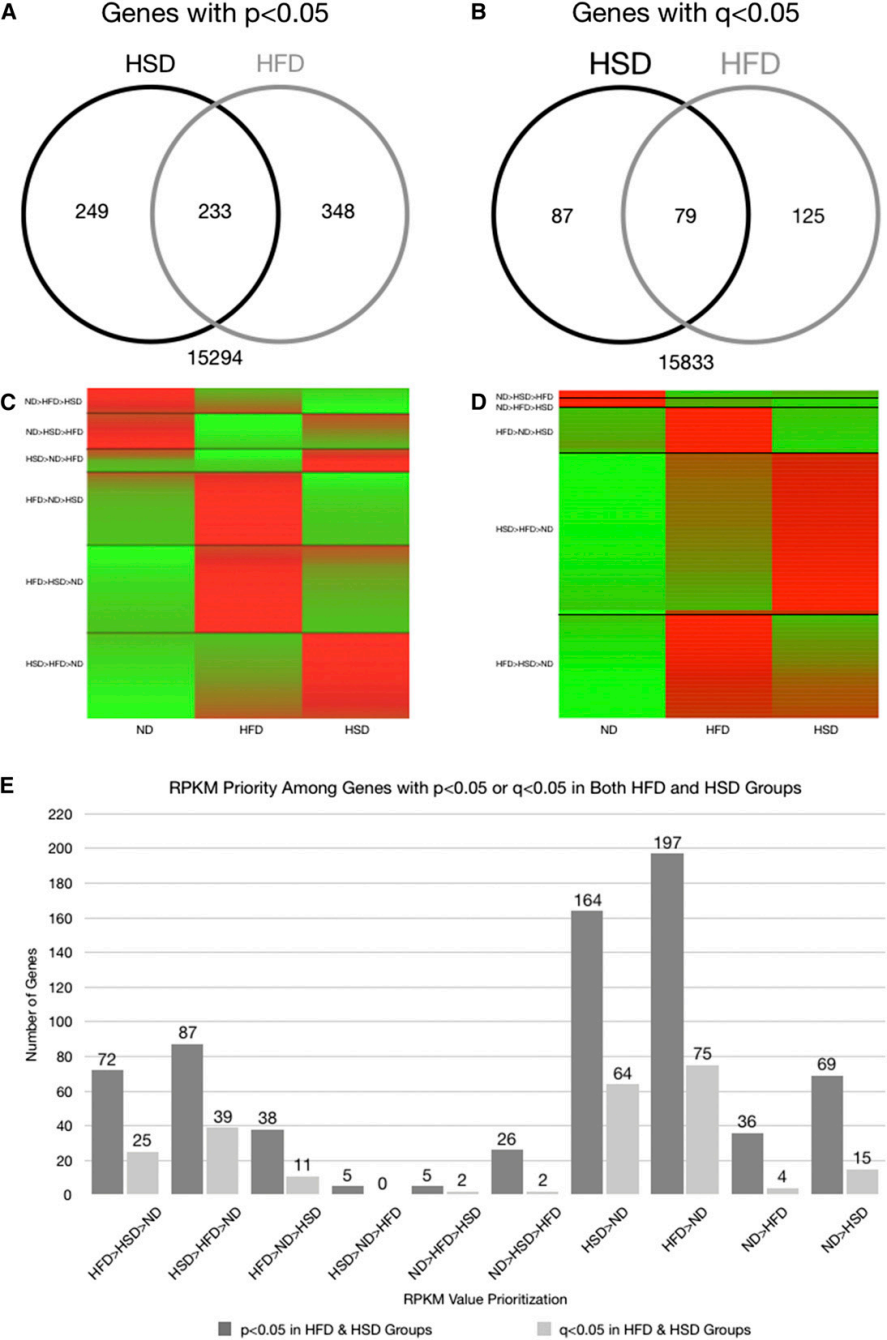
(A) Venn diagram illustrating the number of transcripts with *p* < 0.05 for the HFD, HSD, both diet groups, or neither diet group. (B) Venn diagram illustrating the number of transcripts with *q* < 0.05 in the HFD, HSD, both diet groups, or neither diet group. (C) A heat map of 233 transcripts with *p* < 0.05 in both HSD and HFD groups. The map is divided (black horizontal lines) into six expression priority groups, and, within each group, the transcripts are further arranged by increasing z-score within the HFD column. Bright green indicates the most negative z-score, while bright red indicates the most positive z-score. (D) A heat map of 79 transcripts with *q* < 0.05 in both the HSD and HFD groups. The map is divided (black lines) into five expression priority groups, and, within each group, the transcripts are further arranged by decreasing z-score within the HFD column. Bright green indicates the most negative z-score, while bright red indicates the most positive z-score. (E) A bar graph quantifying the number of genes, among those with *q* < 0.05 or *p* < 0.05 in both obesogenic diet groups, having various RPKM value priorities in the three diet groups.

Counts of genes with significant expression changes by two standards (*q*- and *p*-values) indicate there are over twice as many genes whose expression was significantly affected by a single obesogenic diet, than by both (Figure 1, A and B). Additionally, 11 of the 79 genes whose expression was most significantly affected (*q* < 0.05) were upregulated by the HFD, but downregulated by the HSD, while the opposite was true for no genes (Figure 1, C–E). Curiously, the HFD also upregulated more genes, while the HSD was responsible for the downregulation of more genes, among those significantly affected (Figure 1E and data not shown).

### Functional annotation

The aforementioned genes with significant (*q* < 0.05) expression alterations for at least one obesogenic diet group were further subdivided to produce the gene lists on which to perform functional annotation (described in *Materials and Methods*), and the DAVID Bioinformatics Resources were utilized with suggested defaults to determine the top individual gene annotations and top annotation clusters for each.

**Figure 2:**
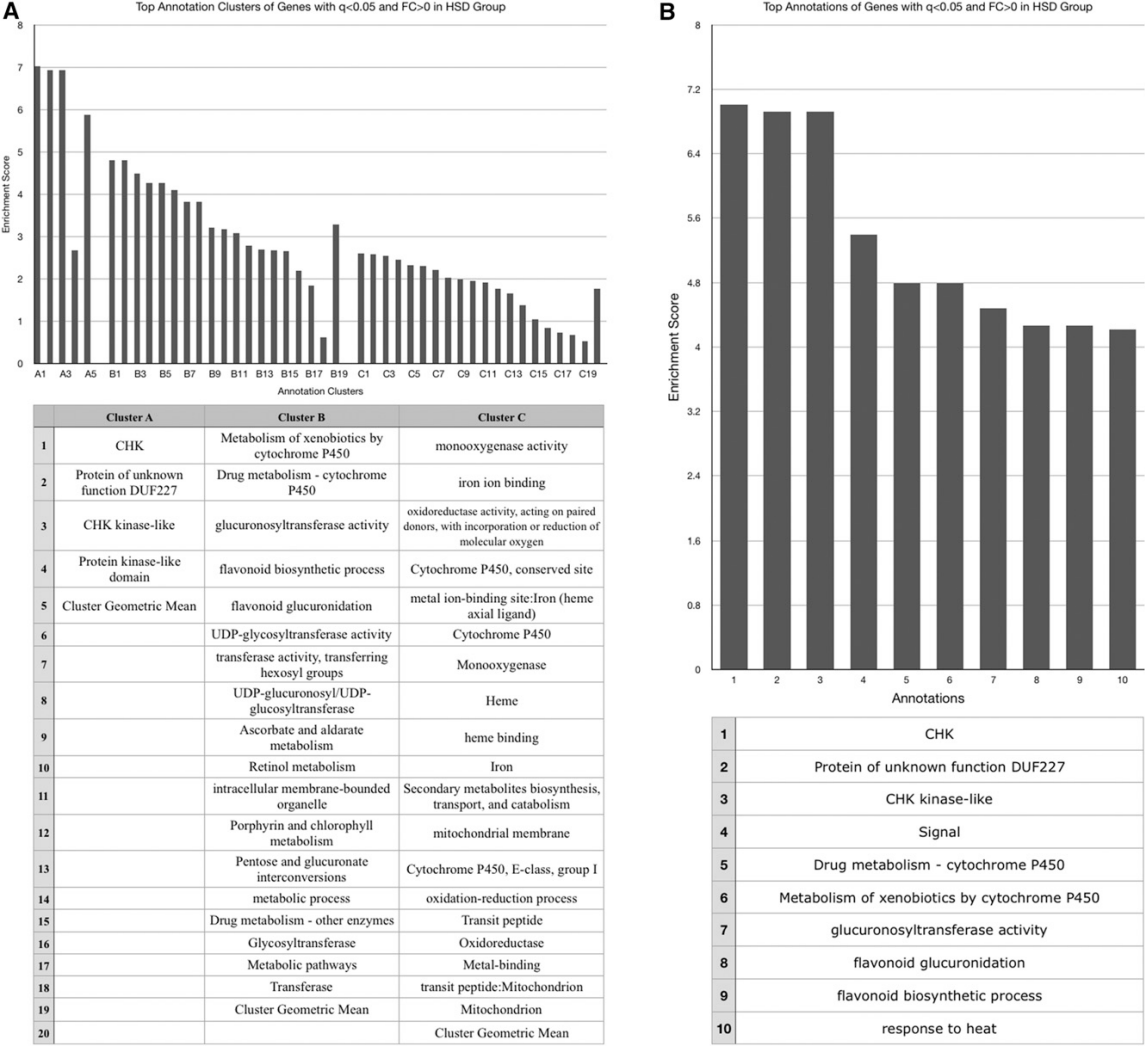
For the cluster graphs (left), bars of the same grouping represent annotations of related functions, belonging to the same cluster (aka annotation clusters), and those bars at the far right of a group represent the enrichment score of the entire cluster (geometric mean of all composing annotations). (A) Graph displaying the three most enriched annotation clusters among genes whose expression was significantly (*q* < 0.05) increased in the HSD group. (B) Graph displaying the most enriched individual annotations among genes whose expression was significantly (*q* < 0.05) increased in the HSD group.

**Figure 3:**
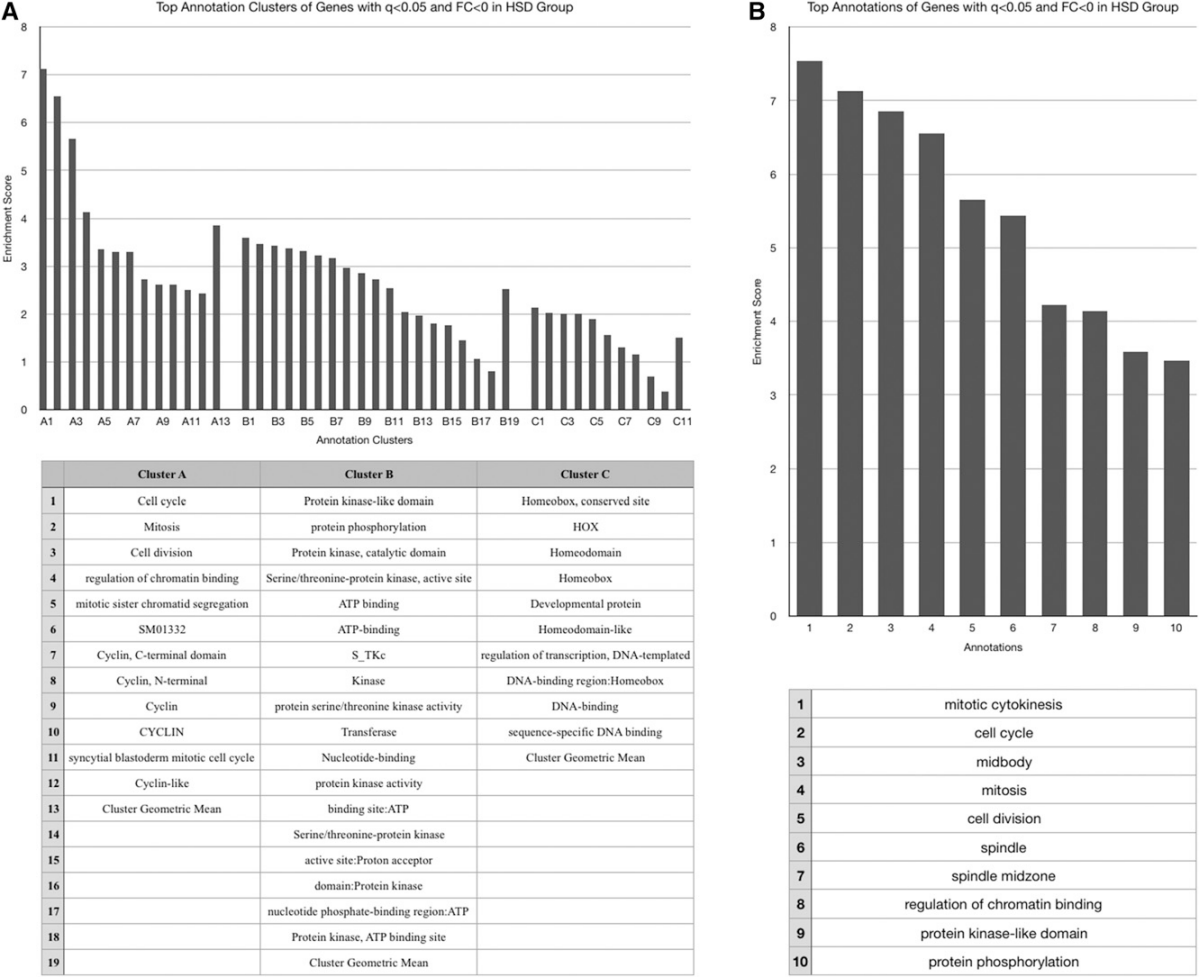
For the cluster graphs (left), bars of the same grouping represent annotations of related functions, belonging to the same cluster (aka annotation clusters), and those bars at the far right of a group represent the enrichment score of the entire cluster (geometric mean of all composing annotations). (A) Graph displaying the three most enriched annotation clusters among genes whose expression was significantly (*q* < 0.05) decreased in the HSD group. (B) Graph displaying the most enriched individual annotations among genes whose expression was significantly (*q* < 0.05) decreased in the HSD group.

**Figure 4:**
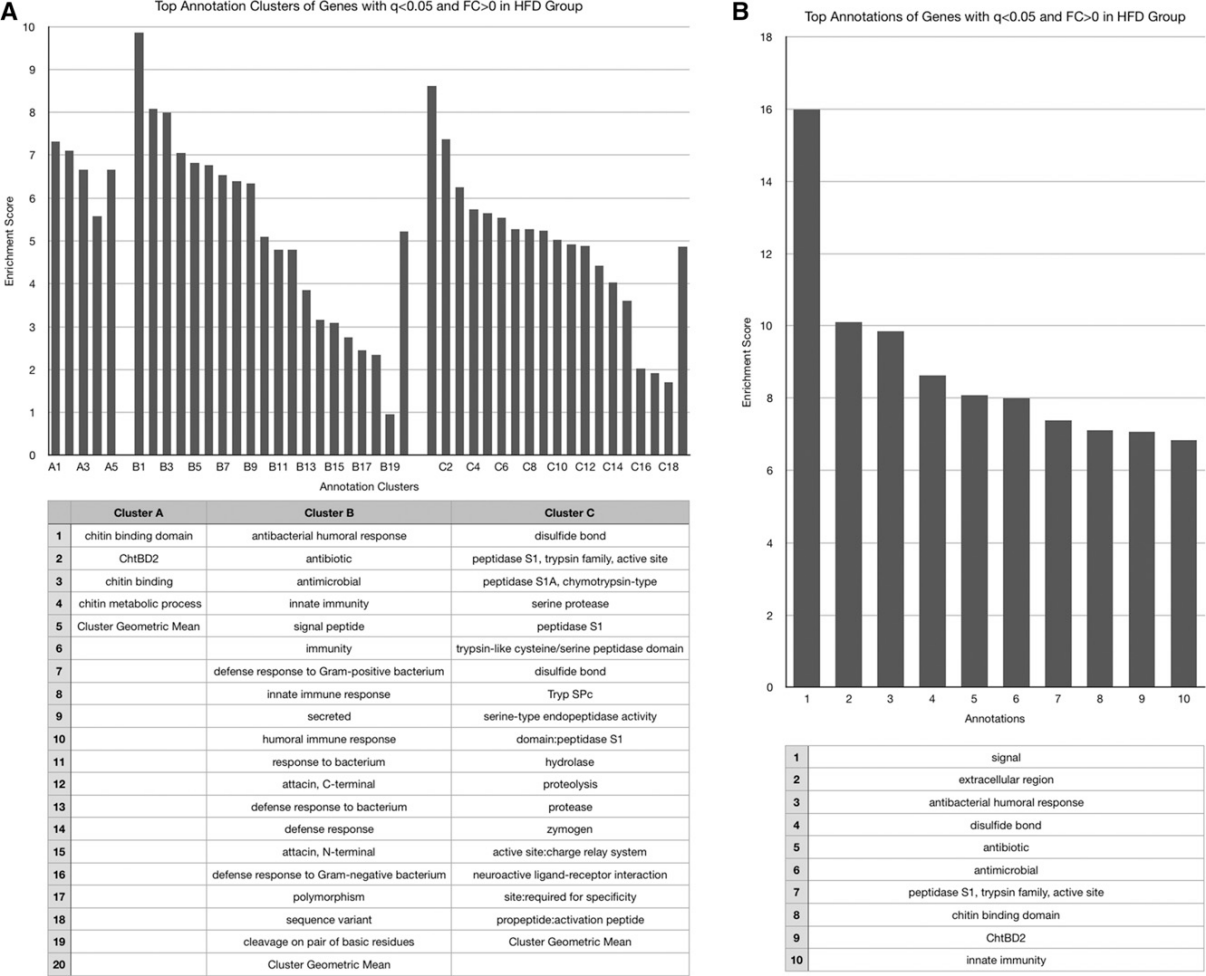
For the cluster graphs (left), bars of the same grouping represent annotations of related functions, belonging to the same cluster (aka annotation clusters), and those bars at the far right of a group represent the enrichment score of the entire cluster (geometric mean of all composing annotations). (A) Graph displaying the three most enriched annotation clusters among genes whose expression was significantly (*q* < 0.05) increased in the HFD group. (B) Graph displaying the most enriched individual annotations among genes whose expression was significantly (*q* < 0.05) increased in the HFD group.

**Figure 5:**
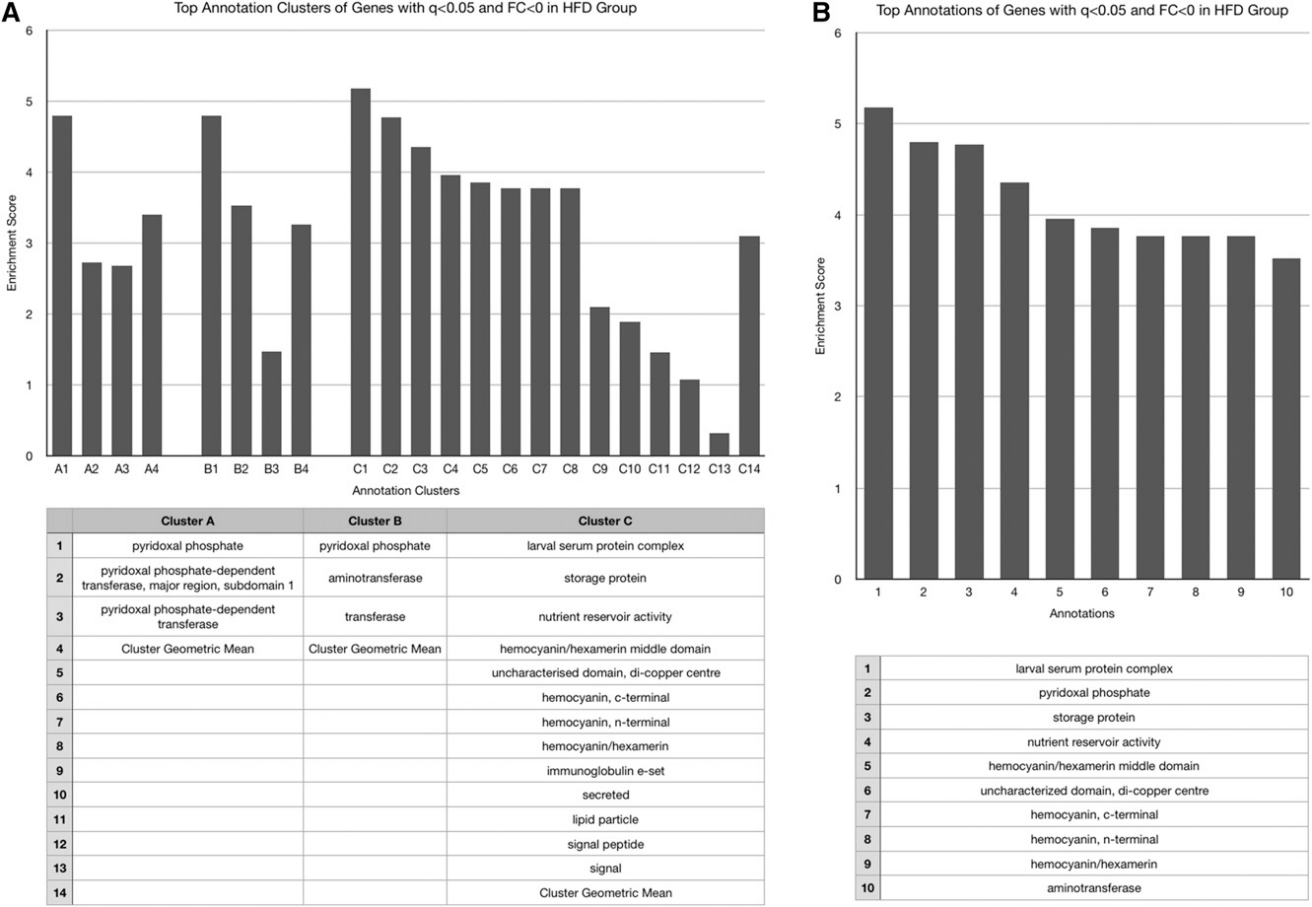
For the cluster graphs (left), bars of the same grouping represent annotations of related functions, belonging to the same cluster (aka annotation clusters), and those bars at the far right of a group represent the enrichment score of the entire cluster (geometric mean of all composing annotations). (A) Graph displaying the three most enriched annotation clusters among genes whose expression was significantly (*q* < 0.05) decreased in the HFD group. (B) Graph displaying the most enriched individual annotations among genes whose expression was significantly (*q* < 0.05) decreased in the HFD group.

**Figure 6:**
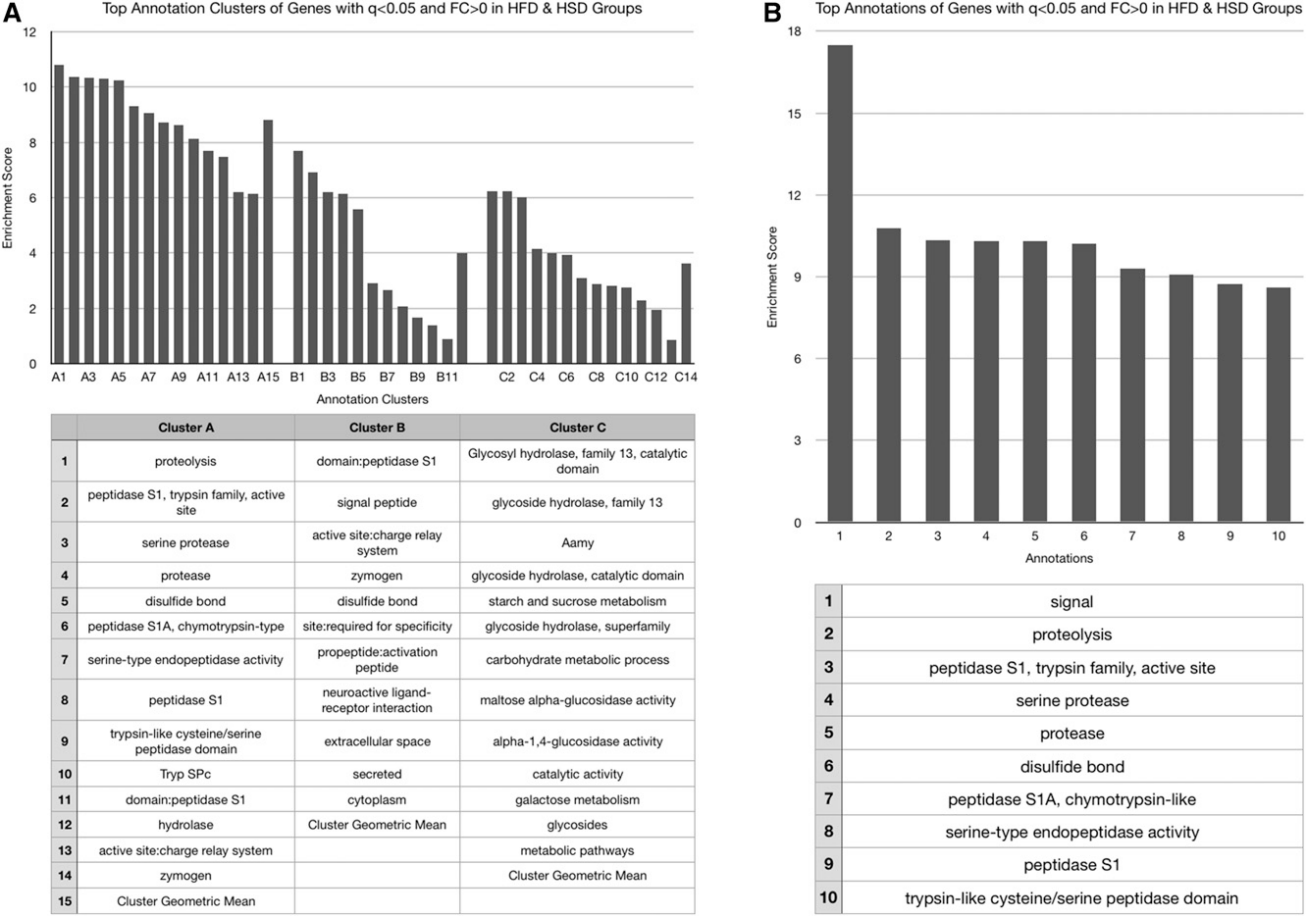
For the cluster graphs (left), bars of the same grouping represent annotations of related functions, belonging to the same cluster (aka annotation clusters), and those bars at the far right of a group represent the enrichment score of the entire cluster (geometric mean of all composing annotations). (A) Graph displaying the three most enriched annotation clusters among genes whose expression was significantly (*q* < 0.05) increased in both obesogenic diet groups. (B) Graph displaying the most enriched individual annotations among genes whose expression was significantly (*q* < 0.05) increased in both obesogenic diet groups.

No functional annotation enrichment data could be gathered from genes whose expression was decreased by both obesogenic diets given the small sample number (*n* = 4). These four genes will be discussed individually later, and are listed in Table 1. The HSD increased expression of genes associated with enzymatic activation, peptide bond cleavage, checkpoint kinases, ascorbate/aldarate and retinol metabolism, cytochrome P450 activity, mitochondrial activity, sugar molecule processing, and heme (Figure 2), and decreased expression of those involved with the cell cycle, kinase activities, cytochrome P450 function, and DNA-binding and transcription (Figure 3). The HFD increased expression of genes with ontology related to immunity and infection, chitin metabolic processing, and protein activities associated with metabolism (Figure 4), and decreased expression of those related to pyridoxal phosphate function, nutrient storage, and signaling, as well as those implicating hemocyanin and immunoglobulins (Figure 5). Genes significantly (*q* < 0.05) upregulated by both obesogenic diets had ontology related to proteolysis, peptide processing, metabolic enzymes, signaling, and carbohydrate metabolism (Figure 6). This indicates gene ontologies that appear characteristic of a HFD (immunity/infection, hemocyanin, and immunoglobulins) or HSD (cytochrome P450 action, CHK, and mitochondrial activity).

**Table 1:** Select genes of functional interest and their associated sequencing and statistical values

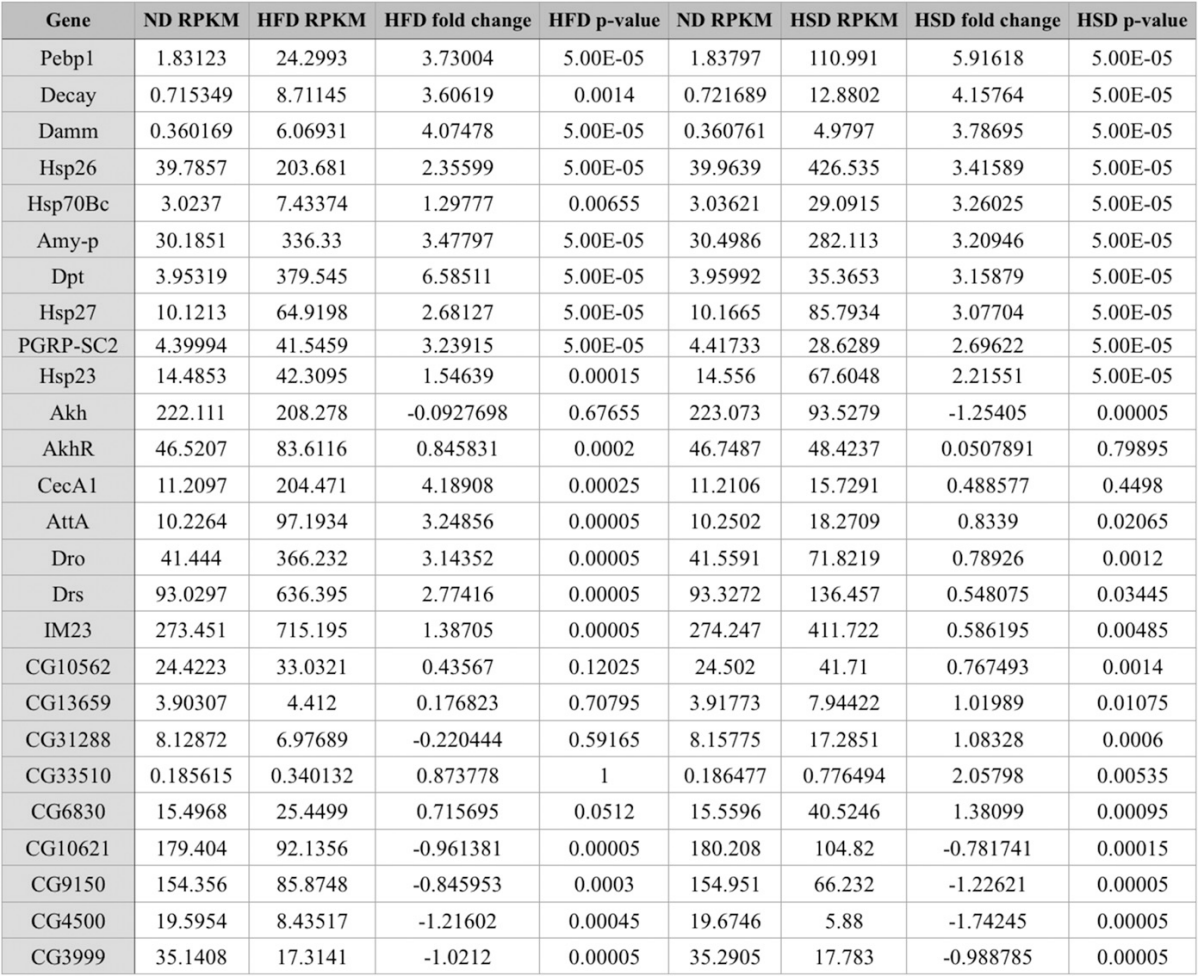

Represented are 10 genes with *q* < 0.05 in both obesogenic diet groups, six genes with *q* < 0.05 in the HFD group only, and six genes with *q* < 0.05 in the HSD group only. Those provided represent the genes included in the most enriched annotation (gene ontology keyword) of relevance (described in *Materials and Methods*) among the genes with *q* < 0.05 in each of three mentioned groups (both diet groups, HFD only, HSD only). Also included are the four genes whose expression was decreased, and had *q* < 0.05, in both obesogenic diet groups; these were absent from the functional annotation results.

### Notable genes

A selection of differentially expressed genes (with *q* < 0.05 in at least one obesogenic diet) with obvious functional relevance were further investigated (Table 1). The remainder of genes not discussed here is publicly available, as noted in *Data availability*.

There was a notable increase in expression of several of the heat shock proteins (in both obesogenic diet groups, except for Hsp70Bc in the HSD group only), which are known to be involved in responses to stress and promotion of longevity in the fly model. There was also increased expression of genes associated with response to bacterial infection, production of antimicrobials, and apoptotic processes, as well as those relating to metabolic regulation. The genes presented for the HSD only list were mostly proteins of unknown function, which were annotated in FlyBase (Gramates *et al.* 2017) as having CHK kinase-like activity, but whose alleged functions were supported by no concrete experimental evidence. Finally, the four genes whose expression was decreased, and had *q* < 0.05, in both obesogenic diet groups contained one gene relating to triglyceride storage and sleep homeostasis, and three genes of unknown biological function.

## Discussion

It is important to re-establish that our findings are within a single genetic background in *D. melanogaster*, using mated females, and utilizing the media recipe variants that we indicated. It is unclear yet how each of these major variables could impact gene expression in these dietary contexts, and we anticipate that further study will elucidate this. It is also important to note that any extrapolation from expression change to a definitive functional impact or physiological mechanism is by nature speculative without direct experimental validation, but a consideration of the implications of our results is necessary. For all genes discussed hereafter, it should be assumed they were significantly affected (*q* < 0.05) in the manner stated, unless otherwise indicated.

### Transcriptome changes

An overview of the transcriptomes of the flies in each dietary group indicates a notable difference in the effects of the two obesogenic diets on gene expression in the fly model. Comparisons between these two obesogenic diet groups’ effects also suggest that the HSD might be more prone to instituting a decrease in gene expression than the HFD, while the HFD might preferentially upregulate gene expression (Figure 1).

### Annotation patterns

Genes with functions related to peptide and carbohydrate processing appear to be the most enriched among genes upregulated by both diets (Figure 6). This is expected, given the increased caloric intake. No functional classes could be ascertained from the four genes noted to be significantly downregulated in both obesogenic diet groups, and these four genes are included in the table of individual genes (Table 1), to be discussed later.

Both diets also upregulated genes functioning in protein cleavage, enzyme activation, and macromolecule processing (Figure 2 and Figure 4). This could be a result of the obesogenic diet-associated macromolecules within the fly, as discussed above. However, the independent enrichment of metabolism related annotations in the HFD- (Figure 4) and HSD- (Figure 2) specific gene ontology lists that are distinct from those enriched in the combined HFD–HSD gene ontology list also implies some distinction in the specific metabolic genes affected. This is unsurprising, given that each diet is enriched with a different macromolecule. These annotations could reflect that, or be an artifact of the DAVID software protocols. However, among those genes upregulated by the HSD, the enriched metabolic processing annotations do appear to relate mostly to sugar/carbohydrate processing (“pentose and glucuronate interconversions,” “transferase activity, transferring hexosyl groups,” and “glycosyltransferase”) (Figure 2).

An enrichment of genes functioning in immunity and response to infection was apparent among genes upregulated by the HFD group (Figure 4). This might imply that the fly is utilizing response mechanisms for the obesogenic diets that appear similar to those used during infection or other immune reactions. This alludes to an overlap in nutrient processing pathways and inflammatory pathways in *Drosophila*, which is supported by the existence of an inflammation-like response during immune reactions in the fly (Shaukat *et al.* 2015).

Among the genes upregulated by the HSD (Figure 2), there was an enrichment of genes relating to checkpoint kinases. Genes coding for CHK kinase-like proteins exist in *Drosophila* (Patil *et al.* 2013), and, in several models, CHK has been established as an arrestor of the cell cycle in response to DNA damage. Additionally, it has the suspected function of indirectly inducing apoptotic mechanisms in response to such damage (Dai and Grant 2010). The increased expression of genes associate with these CHK-related functions in response to the HSD, paired with the decreased expression of genes with functions related to the cell cycle, could imply the ability of diets high in sugar to increase levels of DNA damage and subsequent cell cycle activity impairment. This hypothesis is contributed to by experimental evidence in humans, where obese individuals experience a prolonged increase of free ROS, which are known contributors to DNA damage, following the consumption of a meal high in sugar content (Patel *et al.* 2007).

Among genes downregulated by the HFD, there was enrichment of genes related to hemocyanin (Figure 5), the functional equivalent of hemoglobin in the fly. Recent study has convincingly illustrated the existence of a response to hypoxia in the adipose tissue of obese mammals, and this response may represent a new mechanism for the development of chronic inflammation, macrophage infiltration, insulin resistance, and other detrimental characteristics of an obesity state (Ye 2009). This decrease in expression of genes related to hemocyanin in the HFD group could also imply a relationship between exposure to a HFD and initiation of adipose tissue hypoxia (ATH) in the fly, via the rationale of decreased hemocyanin available for oxygen delivery to adipose tissue. Among genes upregulated by the HSD, those with functions related to heme (major biochemical component of hemoglobin and hemocyanin) processing were enriched, which could suggest a similar ATH state occurring due to the HSD. However, the involvement of heme with a variety of processes in the fly limits definitive conclusion regarding this.

### Genes of interest

Increased expression, in both obesogenic diet groups, of several heat shock proteins suggests that the obesogenic diets may be placing a significant amount of physiological stress on the fly that the Hsps are trying to combat, given the Hsp’s roles in mitigating various types of physiological stress via protein folding and refolding, among other mechanisms (Zhao *et al.* 2010; Vos *et al.* 2016; Azad *et al.* 2009; Wang *et al.* 2004). The Hsps are also identified as having a significant impact on the promotion of longevity in flies, with as much as a 30% increase in lifespan when overexpressed, in contrast to the decreased longevity associated with obesogenic diets (Wang *et al.* 2004; Vos *et al.* 2016). This could imply existence of another mechanism whose effects on lifespan outweigh that of the Hsps. Curiously, activity of Hsp27 has also been associated with an increased resistance to starvation (Hao *et al.* 2007). This seems counterintuitive given the increased caloric intake; however, it is unclear from these data alone whether this function of Hsp27 is active in these flies.

*Akh* is exclusively downregulated by the HSD, and *AkhR* upregulated by the HFD group. Knockdown of *Akh* in flies has produced an increase in triglyceride stores, a decrease in glycogen stores coupled with hypoglycemia in the fly hemolymph, and a decrease in oxidative stress tolerance (Gáliková *et al.* 2015). Downregulation of *Akh* by the HSD is expected for increased metabolite anabolism, and not catabolism, but also might further indicate a potential source of oxidative stress to the fly. In the HFD group, the upregulation of *AkhR* might be promoting the effects of Akh, which is contrary to the expected physiological needs of the fly, and might suggest an avenue of aberrant signaling resulting in excess circulating metabolites. Given these data, the corpora cardiaca could be a fruitful avenue of future directed study into these hypotheses.

There was also upregulation, in both obesogenic diet groups, of two genes known to be involved in apoptotic processes: *Decay* and *Damm* (Harvey *et al.* 2001; Dorstyn *et al.* 1999). Increased expression of *Decay* and *Damm* in our obesogenic diet groups may imply that cell apoptosis is increased in response to obesogenic diets and obesity-like states in *Drosophila*. Overall, more in-depth studies are warranted to investigate these traits in flies on obesogenic diets.

Several genes upregulated in both obesogenic diet groups (*Dpt, PGRP-SC2,* and *Pebp1*) have functions relating to immunity or response to infection (Bischoff *et al.* 2006; Lemaitre *et al.* 1997; Wicker *et al.* 1990; Cronin *et al.* 2009; Reumer *et al.* 2009). Pebp1 binds phosphatidylethanolamine, and overexpression of this gene is associated with protection against both gram-positive and gram-negative bacterial infection in the fly via release of various immunity-related proteins into their hemolymph (Reumer *et al.* 2009). Dpt is noted as being released in varying degrees in response to infections by various types of bacteria, and its overexpression is also established as rescuing flies from the detrimental effects of these infections. Dpt is also released to a lesser extent in response to injury in the fly (Lemaitre *et al.* 1997), a response which often involves release of pro-inflammatory cytokines. Additionally, *Dpt* has been shown to be significantly upregulated in flies that have a tolerance to lifelong hyperoxia, though the significance of this function in the context obesity’s associated detriments is unclear (Wicker *et al.* 1990). PGRP-SC2 enzymatically interacts with peptidoglycan, a major component of bacterial membranes, and provides a specific means by which to protect against bacterial infection (Bischoff *et al.* 2006). It also plays a role in the regulation of the natural microbiota of *Drosophila*, acting to cleave the pro-inflammatory peptidoglycan of their membranes into nonstimulatory muropeptides, and subsequently prevent activation of the fly’s immune system in response to its own endogenous bacteria (Royet *et al.* 2011). There is precedent indicating the ability of diets high in sugar to alter the microbiota of flies in order to compensate for the needed change in metabolic processing (Whon *et al.* 2017), though no direct evidence of this change contributing to an inflammatory state or implicating PGRP-SC2 in the fly exists. Upregulation of these genes in both obesogenic diet groups supports the implication by functional annotation results that flies appear to respond similarly to infections and obesogenic diets (HFD in particular).

Several genes were exclusively upregulated by the HFD, and are established to be involved with immune response in *Drosophila*. These were *CecA1, AttA, Dro, Drs, IM23,* whose expressions are noted to be increased in response to humoral immune system challenge via microbial infection (Lemaitre *et al.* 1997; Uttenweiler-Joseph *et al.* 1998; Verleyen *et al.* 2006; Wagner *et al.* 2009; Levy *et al.* 2004). This diet-specific upregulation of immune related genes reinforces the potential physiologically distinct effects of different obesogenic diets. Ultimately, the large number of immune-associated genes affected by the obesogenic diets implicates hemocytes. This could be the source of these changes, however, infiltration of the fat body by macrophages during obesity-like states has also been established previously (Gregor and Hotamisligil 2011). Both possibilities warrant further study to elucidate this.

Among genes exclusively upregulated by the HSD, five of those related (gene ontology) to CHK kinase-like activity (most enriched annotation) currently have no experimentally supported biological function (Gramates *et al.* 2017). Also, of the four genes noted to be downregulated in both obesogenic diet groups, three had no reported biological function (Gramates *et al.* 2017). However, for one of the four genes, *CG4500,* there exists experimental data involving the knockdown of this gene in *Drosophila*. For these flies, a decrease in stored triglyceride was noted, as well as a negative disruption of sleep homeostasis in the flies studied (Thimgan *et al.* 2015). The suggested effect of obesogenic diets on triglyceride storage (*i.e.*, decreased triglyceride storage via CG4500 decreased expression) is at odds with current direct experimental evidence concerning triglyceride storage in response to HSD and HFD, which could be more evidence of aberrant signaling during an obesity-like state in the fly. The decreased expression of CG4500 could also indicate a disruption of healthy sleep patterns in response to obesogenic diets, in the fly. There exists experimental evidence pointing to the ability of a HSD to selectively affect sleep behavior in *Drosophila* (Catterson *et al.* 2010), as well as, separately, for impaired sleep homeostasis to result in decreased longevity of the fly (Yamazaki *et al.* 2012). Overall, these data emphasize the lack of experimental data relating to genes affected by a HSD, as well as genes downregulated by obesogenic diets, and provide compelling evidence of their potential relevance.

### Conclusions

Data from this research has illustrated a notable effect of obesogenic diets on the transcriptomic profile of *D. melanogaster* head tissue, as well as distinctions in the effects that diets high in sugar *vs.* fat have on gene expression. This data has additionally suggested a variety of physiological functions being activated or suppressed in response to these diets that may provide insight into the development of certain disease states associated with obesity, and indicated specific genes that may play a role in those functions. Overall, these results have provided insight into potential avenues of research to elucidate many of the unknown mechanisms associated with an obesity-like state in *Drosophila*, and provided data with which future investigators may guide their research.

## Supplementary Material

Supplemental material is available online at www.g3journal.org/lookup/suppl/doi:10.1534/g3.117.300397/-/DC1.

## References

[bib1] Azad P. Zhou D. Russo E. Haddad G. G., 2009 Distinct mechanisms underlying tolerance to intermittent and constant hypoxia in *Drosophila melanogaster*. PLoS One 4: e5371. 19401761 10.1371/journal.pone.0005371 PMC2670512

[bib2] Benjamini Y. Hochberg Y., 1995 Controlling the false discovery rate: a practical and powerful approach to multiple testing. J. R. Stat. Soc. B 57: 289–300.

[bib3] Birse R. T. Choi J. Reardon K. Rodriguez J. Graham S., 2010 High fat diet-induced obesity and heart dysfunction is regulated by the TOR pathway in *Drosophila*. Cell Metab. 12: 533–544. 21035763 10.1016/j.cmet.2010.09.014 PMC3026640

[bib4] Bischoff V. Vignal C. Duvic B. Boneca I. G. Hoffmann J. A., 2006 Downregulation of the *Drosophila* immune response by peptidoglycan-recognition proteins SC1 and SC2. PLoS Pathog. 2: e14. 16518472 10.1371/journal.ppat.0020014 PMC1383489

[bib5] Bouleau S. Tricoire H., 2015 *Drosophila* models of Alzheimer’s disease: advances, limits, and perspectives. J. Alzheimers Dis. 45: 1015–1038. 25697708 10.3233/JAD-142802

[bib6] Catterson J. H. Knowles-Barley S. James K. Heck M. M. S. Harmar A. J., 2010 Dietary modulation of *Drosophila* sleep-wake behaviour. PLoS One 5: e12062. 20706579 10.1371/journal.pone.0012062 PMC2919389

[bib7] Cronin S. J. F. Nehme N. T. Limmer S. Liegeois S. Pospisilik J. A., 2009 Genome-wide RNAi screen identifies genes involved in intestinal pathogenic bacterial infection. Science 325: 340–343. 19520911 10.1126/science.1173164 PMC2975362

[bib8] Dai Y. Grant S., 2010 New insights into checkpoint kinase 1 in the DNA damage response signaling network. Clin. Cancer Res. 16: 376–383. 20068082 10.1158/1078-0432.CCR-09-1029 PMC2939735

[bib9] Dennis G. Sherman B. T. Hosack D. A. Yang J. Gao W., 2003 DAVID: database for annotation, visualization, and integrated discovery. Genome Biol. 4: 3. PMC193660 12734009

[bib10] Dorstyn L. Read S. H. Quinn L. M. Richardson H. Kumar S., 1999 DECAY, a novel *Drosophila* caspase related to mammalian caspase-3 and caspase-7. J. Biol. Chem. 274: 30778–30783. 10521468 10.1074/jbc.274.43.30778

[bib11] Feany M. B. Bender W. W., 2000 A *Drosophila* model of Parkinson’s disease. Nature 404: 394–398. 10746727 10.1038/35006074

[bib12] Furukawa S. Fujita T. Shimabukuro M. Iwaki M. Yamada Y., 2004 Increased oxidative stress in obesity and its impact on metabolic syndrome. J. Clin. Invest. 114: 1752–1761. 15599400 10.1172/JCI21625 PMC535065

[bib13] Gáliková M. Diesner M. Klepsatel P. Hehlert P. Xu Y., 2015 Energy homeostasis control in *Drosophila* adipokinetic hormone mutants. Genetics 201: 665–683. 26275422 10.1534/genetics.115.178897 PMC4596676

[bib14] Géminard C. Rulifson E. J. Léopold P., 2009 Remote control of insulin secretion by fat cells in *Drosophila*. Cell Metab. 10: 199–207. 19723496 10.1016/j.cmet.2009.08.002

[bib15] Gramates L. S. Marygold S. J. dos Santos G. Urbano J.-M. Antonazzo G., 2017 FlyBase at 25: looking to the future. Nucleic Acids Res. 45: D663–D671. 27799470 10.1093/nar/gkw1016 PMC5210523

[bib16] Gregor M. F. Hotamisligil G. S., 2011 Inflammatory mechanisms in obesity. Annu. Rev. Immunol. 29: 415–445. 21219177 10.1146/annurev-immunol-031210-101322

[bib17] Hao X. Sen Zhang B. T. Zhang P., 2007 The Hsp27 gene is not required for *Drosophila* development but its activity is associated with starvation resistance. Cell Stress Chaperones 12: 364–372. 18229455 10.1379/CSC-308.1 PMC2134798

[bib18] Harvey N. L. Daish T. Mills K. Dorstyn L. Quinn L. M., 2001 Characterization of the *Drosophila* caspase, DAMM. J. Biol. Chem. 276: 25342–25350. 11337486 10.1074/jbc.M009444200

[bib19] Heinrichsen E. T. Haddad G. G., 2012 Role of high-fat diet in stress response of *Drosophila*. PLoS One 7: e42587. 22870336 10.1371/journal.pone.0042587 PMC3411628

[bib20] Heinrichsen E. T. Zhang H. Robinson J. E. Ngo J. Diop S., 2013 Metabolic and transcriptional response to a high-fat diet in *Drosophila melanogaster*. Mol. Metab. 3: 42–54. 24567903 10.1016/j.molmet.2013.10.003 PMC3929909

[bib21] Khomtchouk B. B. Van Booven D. J. Wahlestedt C., 2014 HeatmapGenerator: high performance RNAseq and microarray visualization software suite to examine differential gene expression levels using an R and C++ hybrid computational pipeline. Source Code Biol. Med. 9: 30. 25550709 10.1186/s13029-014-0030-2 PMC4279803

[bib22] Langdon K. D. Clarke J. Corbett D., 2011 Long-term exposure to high fat diet is bad for your brain: exacerbation of focal ischemic brain injury. Neuroscience 182: 82–87. 21435380 10.1016/j.neuroscience.2011.03.028

[bib23] Lee D. Son H. G. Jung Y. Lee S.-J. V., 2017 The role of dietary carbohydrates in organismal aging. Cell. Mol. Life Sci. 74: 1793–1803. 27942749 10.1007/s00018-016-2432-6 PMC11107617

[bib24] Lehnert T. Sonntag D. Konnopka A. Riedel-Heller S. König H.-H., 2013 Economic costs of overweight and obesity. Best Pract. Res. Clin. Endocrinol. Metab. 27: 105–115. 23731873 10.1016/j.beem.2013.01.002

[bib25] Lemaitre B. Reichhart J. M. Hoffmann J. A., 1997 *Drosophila* host defense: differential induction of antimicrobial peptide genes after infection by various classes of microorganisms. Proc. Natl. Acad. Sci. U S A 94:14614–14619. 9405661 10.1073/pnas.94.26.14614 PMC25070

[bib26] Levy F. Rabel D. Charlet M. Bulet P. Hoffmann J. A., 2004 Peptidomic and proteomic analyses of the systemic immune response of *Drosophila*. Biochimie 86: 607–616. 15556270 10.1016/j.biochi.2004.07.007

[bib27] Lewis S. B. Wallin J. D. Kane J. P. Gerich J. E., 1977 Effect of diet composition on metabolic adaptations to hypocaloric nutrition: comparison of high carbohydrate and high fat isocaloric diets. Am. J. Clin. Nutr. 30: 160–170. 835502 10.1093/ajcn/30.2.160

[bib28] Lin S. Thomas T. C. Storlien L. H. Huang X. F., 2000 Development of high fat diet-induced obesity and leptin resistance in C57Bl/6J mice. Int. J. Obes. Relat. Disord. 24: 639–646. 10.1038/sj.ijo.0801209 10849588

[bib29] McNay E. C. Ong C. T. McCrimmon R. J. Cresswell J. Bogan J. S., 2010 Hippocampal memory processes are modulated by insulin and high-fat-induced insulin resistance. Neurobiol. Learn. Mem. 93: 546–553. 20176121 10.1016/j.nlm.2010.02.002 PMC2878207

[bib30] Montague C. T. Farooqi I. S. Whitehead J. P. Soos M. A. Rau H., 1997 Congenital leptin deficiency is associated with severe early-onset obesity in humans. Nature 387: 903–908. 9202122 10.1038/43185

[bib31] Musselman L. P. Fink J. L. Narzinski K. Ramachandran P. V. Hathiramani S. S., 2011 A high-sugar diet produces obesity and insulin resistance in wild-type *Drosophila*. Dis. Model. Mech. 4: 842–849. 21719444 10.1242/dmm.007948 PMC3209653

[bib32] Must A. Spadano J. Coakley E. H. Field A. E. Colditz G., 1999 The disease burden associated with overweight and obesity. JAMA 282: 1523–1529. 10546691 10.1001/jama.282.16.1523

[bib33] Na J. Musselman L. P. Pendse J. Baranski T. J. Bodmer R., 2013 A *Drosophila* model of high sugar diet-induced cardiomyopathy. PLoS Genet. 9: e1003175. 23326243 10.1371/journal.pgen.1003175 PMC3542070

[bib34] Patel C. Ghanim H. Ravishankar S. Sia C. L. Viswanathan P., 2007 Prolonged reactive oxygen species generation and nuclear factor-kappaB activation after a high-fat, high-carbohydrate meal in the obese. J. Clin. Endocrinol. Metab. 92: 4476–4479. 17785362 10.1210/jc.2007-0778

[bib35] Patil M. Pabla N. Dong Z., 2013 Checkpoint kinase 1 in DNA damage response and cell cycle regulation. Cell Mol. Life Sci. 70: 4009–4021. 23508805 10.1007/s00018-013-1307-3 PMC3731415

[bib36] Piya M. K. McTernan P. G. Kumar S., 2013 Adipokine inflammation and insulin resistance: the role of glucose, lipids and endotoxin. J. Endocrinol. 216: T1–T15. 23160966 10.1530/JOE-12-0498

[bib37] Reiter L. T. Potocki L. Chien S. Gribskov M. Bier E., 2001 A systematic analysis of human disease-associated gene sequences in *Drosophila melanogaster*. Genome Res. 11: 1114–1125. 11381037 10.1101/gr.169101 PMC311089

[bib38] Reumer A. Bogaerts A. Van Loy T. Husson S. J. Temmerman L., 2009 Unraveling the protective effect of a *Drosophila* phosphatidylethanolamine-binding protein upon bacterial infection by means of proteomics. Dev. Comp. Immunol. 33: 1186–1195. 19545586 10.1016/j.dci.2009.06.010

[bib39] Rodríguez A. Ezquerro S. Méndez-Giménez L. Becerril S. Frühbeck G., 2015 Revisiting the adipocyte: a model for integration of cytokine signaling in the regulation of energy metabolism. Am. J. Physiol. Endocrinol. Metab. 309: E691–E714. 26330344 10.1152/ajpendo.00297.2015

[bib40] Rovenko B. M. Kubrak O. I. Gospodaryov D. V. Perkhulyn N. V. Yurkevych I. S., 2015 High sucrose consumption promotes obesity whereas its low consumption induces oxidative stress in *Drosophila melanogaster*. J. Insect Physiol. 79: 42–54. 26050918 10.1016/j.jinsphys.2015.05.007

[bib41] Royet J. Gupta D. Dziarski R., 2011 Peptidoglycan recognition proteins: modulators of the microbiome and inflammation. Nat. Rev. Immunol. 11: 837–851. 22076558 10.1038/nri3089

[bib42] Shaukat Z. Liu D. Gregory S., 2015 Sterile inflammation in *Drosophila*. Mediators Inflamm. 2015: 369286. 25948885 10.1155/2015/369286 PMC4408615

[bib43] Shih J. Hodge R. Andrade-Navarro M. A., 2014 Comparison of inter- and intraspecies variation in humans and fruit flies. Genom. Data 3: 49–54. 26484147 10.1016/j.gdata.2014.11.010 PMC4536057

[bib44] Stranahan A. M. Mattson M. P., 2011 Bidirectional metabolic regulation of neurocognitive function. Neurobiol. Learn. Mem. 96: 507–516. 21236352 10.1016/j.nlm.2011.01.004 PMC3084367

[bib45] Thimgan M. S. Seugnet L. Turk J. Shaw P. J., 2015 Identification of genes associated with resilience/vulnerability to sleep deprivation and starvation in *Drosophila*. Sleep 38: 801–814. 25409104 10.5665/sleep.4680 PMC4402663

[bib46] Trapnell C. Williams B. A. Pertea G. Mortazavi A. Kwan G., 2010 Transcript assembly and abundance estimation from RNA-seq reveals thousands of new transcripts and switching among isoforms. Nat. Biotechnol. 28: 511–515. 20436464 10.1038/nbt.1621 PMC3146043

[bib47] Uttenweiler-Joseph S. Moniatte M. Lagueux M. Van Dorsselaer A. Hoffmann J. A., 1998 Differential display of peptides induced during the immune response of *Drosophila*: a matrix-assisted laser desorption ionization time-of-flight mass spectrometry study. Proc. Natl. Acad. Sci. USA 95: 11342–11347. 9736738 10.1073/pnas.95.19.11342 PMC21644

[bib48] Valladolid-Acebes I. Stucchi P. Cano V. FernÁndez-Alfonso M. S. Merino B., 2011 High-fat diets impair spatial learning in the radial-arm maze in mice. Neurobiol. Learn. Mem. 95: 80–85. 21093599 10.1016/j.nlm.2010.11.007

[bib49] Verleyen P. Baggerman G. D’Hertog W. Vierstraete E. Husson S. J., 2006 Identification of new immune induced molecules in the haemolymph of *Drosophila* *melanogaster* by 2D-NanoLC MS/MS. J. Insect Physiol. 52: 379–388. 16510152 10.1016/j.jinsphys.2005.12.007

[bib50] Vos M. J. Carra S. Kanon B. Bosveld F. Klauke K., 2016 Specific protein homeostatic functions of small heat-shock proteins increase lifespan. Aging Cell 15: 217–226. 26705243 10.1111/acel.12422 PMC4783350

[bib51] Wagner C. Isermann K. Roeder T., 2009 Infection induces a survival program and local remodeling in the airway epithelium of the fly. FASEB J. 23: 2045–2054. 19237508 10.1096/fj.08-114223

[bib52] Wang H.-D. Kazemi-Esfarjani P. Benzer S., 2004 Multiple-stress analysis for isolation of *Drosophila* longevity genes. Proc. Natl. Acad. Sci. USA 101: 12610–12615. 15308776 10.1073/pnas.0404648101 PMC515105

[bib53] Whon T. W. Shin N. R. Jung M. J. Hyun D. W. Kim H. S., 2017 Conditionally pathogenic gut microbes promote larval growth by increasing redox-dependent fat storage in high-sugar diet-fed *Drosophila*. Antioxid. Redox Signal. 27: 1361–1380. 28462587 10.1089/ars.2016.6790

[bib54] Wicker C. Reichhart J. M. Hoffmann D. Hultmark D. Samakovlis C., 1990 Insect immunity. Characterization of a *Drosophila* CDNA encoding a novel member of the diptericin family of immune peptides. J. Biol. Chem. 265: 22493–22498. 2125051

[bib55] Xu H. Barnes G. T. Yang Q. Tan G. Yang D., 2003 Chronic inflammation in fat plays a crucial role in the development of obesity-related insulin resistance. J. Clin. Invest. 112: 1821–1830. 14679177 10.1172/JCI19451 PMC296998

[bib56] Yamazaki M. Tomita J. Takahama K. Ueno T. Mitsuyoshi M., 2012 High calorie diet augments age-associated sleep impairment in *Drosophila*. Biochem. Biophys. Res. Commun. 417: 812–816. 22197809 10.1016/j.bbrc.2011.12.041

[bib57] Ye J., 2009 Emerging role of adipose tissue hypoxia in obesity and insulin resistance. Int. J. Obes. 33: 54–66. 10.1038/ijo.2008.229 PMC2650750 19050672

[bib58] Zhao H. W. Zhou D. Nizet V. Haddad G. G., 2010 Experimental selection for *Drosophila* survival in extremely high O2 environments. PLoS One 5: e11701. 20668515 10.1371/journal.pone.0011701 PMC2909141

