# Supplementary material for: RNA-Sequencing of Drosophila melanogaster Head Tissue on High-Sugar and High-Fat Diets
Source: G3 (Bethesda). 2017 Nov 15;8(1):279–90. doi: 10.1534/g3.117.300397 (PMC5765356; doi:10.1534/g3.117.300397)
Supplement: Supplementary file 1 [file 279FileS3.docx]

**RNA-Sequencing Quality Control Information**

*Methodology*

Twelve RNA samples (4 replicates from each of 3 dietary treatment group) of the highest yield (>100 ng/µL) and sufficient purity (260/280 and 260/230 ratios of 1.8-2.1 and >1.5, respectively), ascertained via NanoDrop^®^ 2000c (Thermo Scientific) readings, were sent to Louisiana State University Health Science Center in Shreveport for initial Poly-A selected RNA-sequencing in the Genomics Core Facility. mRNA was isolated from the RNA via poly-adenylated RNA selection using oligomer beads, and subsequently fragmented, before reverse transcription into cDNA and attachment of the adapter sequences and other necessary motifs for sequencing. They prepared the cDNA libraries using a TruSeq Stranded mRNA Kit (Illumina). The created cDNA libraries were normalized, denatured, and diluted, and a 1% library of 1.8 pM PhiX Control v3 (Illumina) was added to each to act as an internal control. PhiX functions as a reference for the number of clusters produced by samples, provides a template for calculating sequencing error, and contributes to the diversity of the sequence samples to assist with sequenced transcript discrimination. An Agilent Tape Station 2200 D1000 assay was subsequently performed to measure the fragment size range of our cDNA libraries prepared from the isolated RNA, confirming appropriate fragment sizes prior to sequencing. Samples were then sequenced using a NextSeq 500 (Illumina), targeting at least 50 million stranded, paired end reads of 75 base pairs in size. After subsequent sequencing of the cDNA, the raw data was then passed through a software filter (RTA v2.4.6.0) to remove sub-optimum quality reads (i.e. reads with low confidence in their composing base-calls, and/or the identity of the associated gene), a gauge of implemented read quality was performed (≥Q30), and total read number for each sample was obtained. The ≥Q30 value indicates the percent of base calls in the sequence who have less than a 0.1% chance of being Type I errors. Following raw data production and initial filtering with Illumina RTA software, data was sent to the laboratory of Dr. Urska Cvek at Louisiana State University at Shreveport for further analysis to ascertain reads per million, differential expression analysis between the three conditions, and the identity of sequenced transcripts via mapping against the Drosophila mRNA records using Cufflinks. Transcript value (RPKM) distributions for replicates of each condition were normalized (for each obesogenic diet group relative to the ND group) to produce fold change values, and p-values (representing the significance of the differences between the obesogenic and normal diet groups’ distributions) were calculated using Cuffdiff (Cufflinks tool).

*Results*

The TapeStation results illustrate a fragmentation pattern of the mRNA that produced cDNA fragment sizes concentrated between 200-400 bp in size, in a manner that was consistent across samples (Figure 1A). Quantitation of the average fragment size further supports the consistency of the fragment sizes across the samples, with the average transcript sizes for each of the samples ranging from 301-313 base pairs (Figure 1B). The high percent of reads from each lane of the flow cell noted as passing the software filter indicates that the preparation and sequencing were of high quality, having required few reads be discarded (Figure 2B). The high, comparable number of paired-end reads from each sample passing the software filter (54.6 - 74.1 million) indicates that the RPKM values later generated will have a large enough total read basis to promote statistical significance, and that this degree of significance will be relatively consistent across samples (Figure 2C). Precedent has indicated that approximately 10-fold genome coverage of a desired organism’s sequencing is sufficient for generating reliable RPKM values measuring the expression of all targeted transcripts, including those of low relative abundance, as well as all splice variants. FlyBase indicates that the Drosophila melanogaster genome is 102,160, 260 base pairs in length (17730 genes at an average of 5762 bp), thus, a successful sequence would cover 1,021,602,600 bp worth of reads. For a sequence with average transcript sizes of ~300bp, like this one, each sample read would need to be a minimum of ~3.5 million reads to have reliable data. Our data illustrates a minimum read number of >50 million reads for each sample (Figure 2C), indicating a basis for reliable sequencing results. A high percent of reads with a quality index score greater than or equal to 30 (≥Q30) shows that the majority of reads accepted for mapping were of high base-calling confidence, and therefore transcript identity reliability. The large percent of clusters (identical, sequenced transcripts) passing the software filter additionally supports the quality of the reads and sequencing, and furthermore, indicates this quality was prevalent across the various transcripts sequenced (Figure 2A).


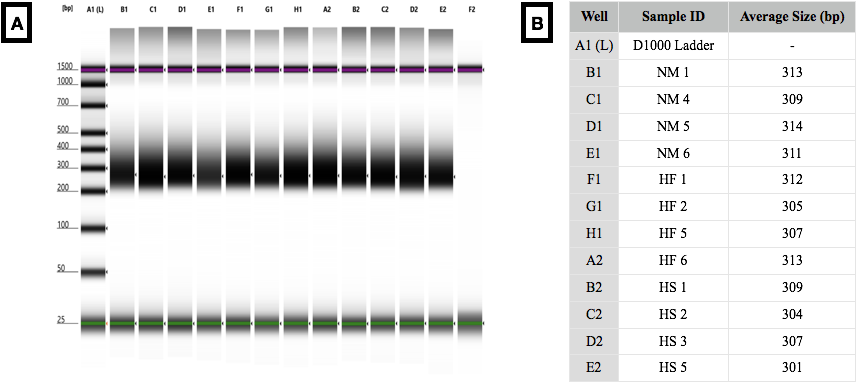


*Figure 1*: (A) Image of Agilent TapeStation 2200 D100 assay results. Bands marked with a purple or green line indicate fragments generated due to the presence of the 1% library of 1.8 pM PhiX control. On the far left is a DNA ladder included to act as a size standard. The size of the standard bands in base pairs is indicated at the left of each band. (B) A table indicating the specific samples corresponding to each lane of the D1000 assay, and containing the average size in base pairs of the fragments present in each sample. For the column labeled “Sample ID”, those identities included refer to the RNA extracted from a group of flies reared on either a ND (NM), HFD (HF), or HSD (HS), with the following number distinguishing between replicate groups of flies reared on the same diet type.


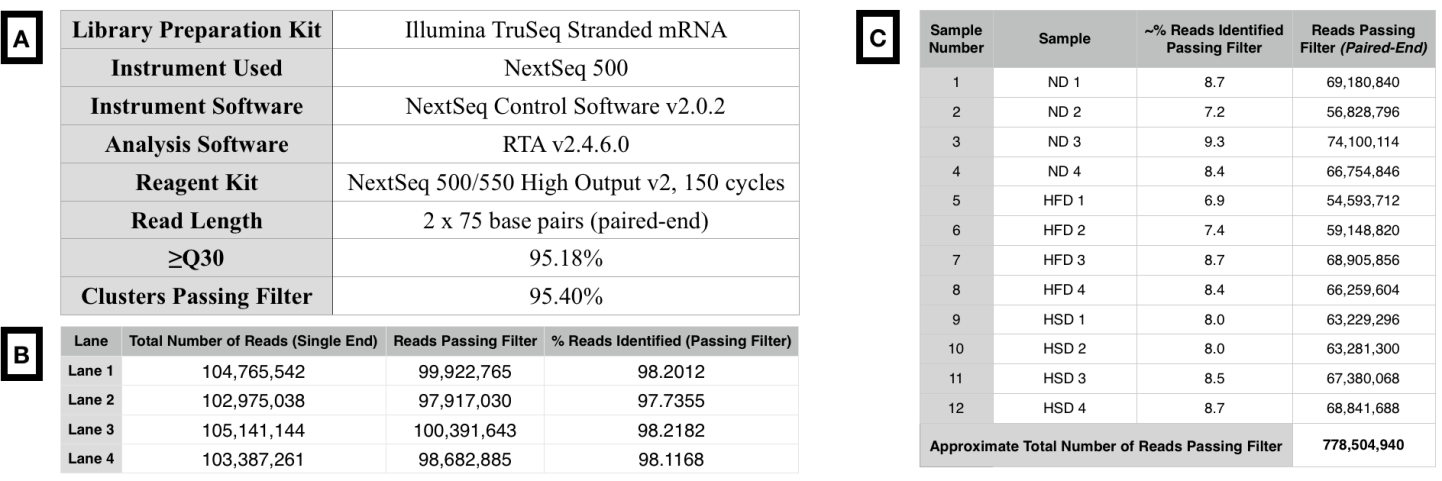


*Figure 2*: (A) An overview of the sequencing and quality control information. (B) Table showing the number of reads noted in each flow cell lane, and the number and percent of those reads passing the software filter. (C) Table showing the number and percent of reads passing the software filter, as contributed by each sample. Samples shown represent the four sequenced fly group replicates for each diet type.
